# Validation of clinical-radiological scores for prognosis of mortality in acute pulmonary embolism

**DOI:** 10.1186/s12931-023-02489-0

**Published:** 2023-08-05

**Authors:** Alexey Surov, Maximilian Thormann, Caroline Bär, Andreas Wienke, Jan Borggrefe

**Affiliations:** 1 Department of Radiology, Neuroradiology and Nuclear Medicine, Johannes Wesling University Hospital, Ruhr University Bochum, Minden, Germany; 2 Department of Radiology and Nuclear Medicine, Otto-von-Guericke University Magdeburg, Leipziger Str. 44, 39120 Magdeburg, Germany; 3 Institute of Medical Epidemiology, Biostatistics, and Informatics, Martin-Luther- University Halle-Wittenberg, Halle, Germany

**Keywords:** Acute pulmonary embolism, Mortality, Computed tomography

## Abstract

**Introduction:**

Acute pulmonary embolism (APE) is a hazardous disorder with a high mortality. Combination of clinical, radiological, and serological parameters can improve risk stratification of APE. Most of the proposed combined scores were not validated in independent cohorts. Our aim was to validate the proposed clinical-radiological scores for prognosis of 7- and 30-day mortality in APE.

**Materials and methods:**

Our sample comprised 531 patients with APE, mean age 64.8 ± 15.6 years, 221 (41.6%) females and 310 (58.4%) males. The following parameters were collected: Age and sex of the patients, mortality within the observation time of 30 days, simplified pulmonary embolism severity index (sPESI), pH troponin level (pg/ml), minimal systolic and diastolic blood pressures (mmHg), heart rate, O_2_ saturation, episodes of syncope, and need for vasopressors. On CT pulmonary angiography (CTPA), short axis ratio right ventricle/left ventricle (RV/LV), and reflux of contrast medium into the inferior vena cava were obtained. The following clinical-radiological scores were calculated: BOVA score, pulmonary embolism mortality score (PEMS), European Society of Cardiology (ESC) score, Kumamaru score, and Calgary acute pulmonary embolism (CAPE) score.

**Results:**

Overall, 31 patients (5.8%) died within seven and 64 patients (12%) within 30 days. All scores showed high negative prognostic values ranging from 89.0 to 99.0%. PEMS and CAPE score demonstrated the highest specificity for 7-day mortality (93.4% and 85.0%), PEMS and BOVA for 30-day mortality (94.2% and 90.4%). The highest sensitivity was observed for ESC 2019 (96.8% and 95.3%). Kumamaru and CAPE scores had low sensitivity. All scores had low positive and high negative predictive values.

**Conclusion:**

For prognosis of 7- and 30-day mortality in APE, PEMS score has the highest specificity. ESC 2019 shows the highest sensitivity. All scores had low positive and high negative predictive values.

## Introduction

Acute pulmonary embolism (APE) is a hazardous condition with a high mortality rate [1, 2]. Therefore, a prompt diagnosis of APE is crucial. The current gold standard for the diagnosis of APE is computer tomographic pulmonary angiography (CTPA). Additionally, an immediate risk assessment of patients with APE upon presentation is important. Various clinical, serological, and imaging factors have been found to have prognostic value in predicting outcomes for patients with APE. For instance, elevated troponin levels independently contribute to the prognosis of short-term and long-term outcomes [2]. Similarly, serum lactate levels serve as a prognostic factor for short-term complications related to pulmonary embolism [3]. Patients with high lactate values have a higher mortality rate [3]. Furthermore, several clinical parameters have been identified as significant prognostic factors in APE. Survivors tend to have higher mean and systolic arterial pressures compared to non-survivors [4]. In terms of imaging, different CTPA parameters hold prognostic value for morbidity and/or mortality in patients with APE [5–7]. For instance, increased ratio of right ventricle diameter to left ventricle diameter (RV/LV ratio) measured on transverse CTPA images is associated with a high risk for all-cause mortality [7]. Contrast reflux in to the inferior vena cava is another strong prognostic factor for short-term mortality in patients with APE [6].

Several studies suggest that combining radiological, clinical, and serological parameters can enhance the risk assessment of APE patients [8–12]. The reported combined scores demonstrate high diagnostic value [8–12]. However, most of the proposed scores lack validation on independent cohorts, leaving the true prognostic potential of these scores uncertain.

The objective of the present study was to validate the previously proposed combined clinical-radiological scores, as reported in the literature, for predicting 7- and 30-day mortality in APE. The validation was conducted using a large cohort of patients.

## Methods

This retrospective study was approved by the institutional review board (number: 58/22, Ethic Commission of the Medical Faculty, Otto-von-Guericke University Magdeburg).

### Patients

For the current study, a screening of the electronic databases of the radiological department was conducted, encompassing the time period from 2015 to 2021. All patients diagnosed with acute pulmonary embolism were extracted from the database. In total, 571 patients were identified as meeting the criteria for inclusion in the study. The inclusion criteria were as follows:

- Age ≥ 18 years.
- Evidence of APE on CTPA.
- Available CTPA images in the picture archiving and communication system (PACS).
- Available clinical data (age and gender, minimal systolic and diastolic blood pressures, heart rate, episodes of syncope, and need for vasopressors).
- Available biochemical data including pH, O_2_ saturation, and troponin.

Exclusion criteria were:

- Incomplete visualization of pulmonary arteries on CT scan.
- Chronic PE.

Clinical data was collected immediately before or after CT scan.

Follow-up was performed by electronic hospital charts. Overall, 40 patients were excluded. Our sample comprised 531 patients with APE, 221 (41.6%) females and 310 (58.4%) males, mean age 64.8 ± 15.6 years, median age 66 years. Baseline demographics and clinical characteristics are given in Table 1.

**Table 1:** Patients demographics and scores

Characteristics	survivors* (n = 467)	Non-survivors* (n = 64)	*p*-value
Age, years	64.3 ± 15,9; (65)	68.6 ± 12,2 (69)	0.062
Age > 80, years	n = 74 (15%)	n = 10 (16%)	0.964
male	n = 264 (57%)	n = 46 (72%)	0.020
female	n = 203 (43%)	n = 18 (28%)	0.020
Known malignancy	n = 140 (30%)	n = 31 (48%)	0.003
Chronic heart failure	n = 66 (14%)	n = 15 (23%)	0.052
Chronic pulmonary disease	n = 90 (19%)	n = 14 (22%)	0.623
Chronic heart failure and/or chronic pulmonary disease	n = 137 (29%)	n = 21 (33%)	0.568
RV Diameter [mm], median (range)	40 (34–47)	39 (33–49)	0.861**
LV Diameter [mm], median (range)	38 (32–44)	37 (30–44)	0.557**
RV-/LV-Ratio, median (range)	1.00 ( 0,84–1,30)	1.04 (0,85–1,36)	0.471**
IVC-Reflux grade 0	n = 169 (36%)	n = 23 (36%)	0.969
IVC-Reflux grade I	n = 163 (35%)	n = 21 (33%)	0.742
IVC-Reflux grade II	n = 112 (24%)	n = 14 (22%)	0.710
IVC-Reflux grade III	n = 23 (5%)	n = 6 (9%)	0.142
Pleural effusion	n = 175 (38%)	n = 44 (69%)	< 0,001
Pericardial effusion	n = 35 (8%)	n = 14 (22%)	< 0.001
Ascites	n = 48 (10%)	n = 21 (33%)	< 0.001
hs TnT [ng/ml], median (range)	0.0320 _(0.0137 – 0.0802)_	0.0805 _(0.0382 – 0.2272)_	< 0.001**
hs TnT > 0,039ng/ml	n = 137 (29%)	n = 29 (45%)	0.010
Need for vasopressors	n = 33 (7%)	n = 24 (38%)	< 0,001
Central APE	n = 156 (33%)	n = 21 (33%)	0.925
Sub-/Segmental APE	n = 311 (67%)	n = 43 (67%)	0.925
sPESI, median (range)	1 (0–2)	2 (1–2)	< 0.001
BOVA Score, median (range)	2 (0–3)	3 (1–4)	0.011
PEMS, median (range)	0 (0–1)	1 (1–3)	< 0.001
Kumamaru Score, median (range)	85 (68–112)	122 (92–159)	< 0.001
ESC guidelines 2019, median (range)	2 (2–3)	3 (2–3)	< 0.001
CAPE Score, median (range)	1 (0–2)	1 (1–2)	0.016

* rates are reported for 30-day mortality

In all cases, the diagnosis of APE was confirmed using CTPA. The CTPA scans were performed on multi-slice CT scanners, specifically Siemens Somatom Definition AS+ (Siemens Healthcare, Germany) or Canon Aquilion Prime (Canon Medical Systems, Ottawara, Japan). To enhance the visibility of the blood vessels, an iodinated contrast agent was administered intravenously via a peripheral venous line at a rate of 3.0–4.0 ml/s. Automatic bolus tracking was utilized in the pulmonary trunk, with a trigger set at 100 Hounsfield units (HU) to initiate the scanning process. All patients received non-invasive medical treatment for APE.

**Fig. 1:**
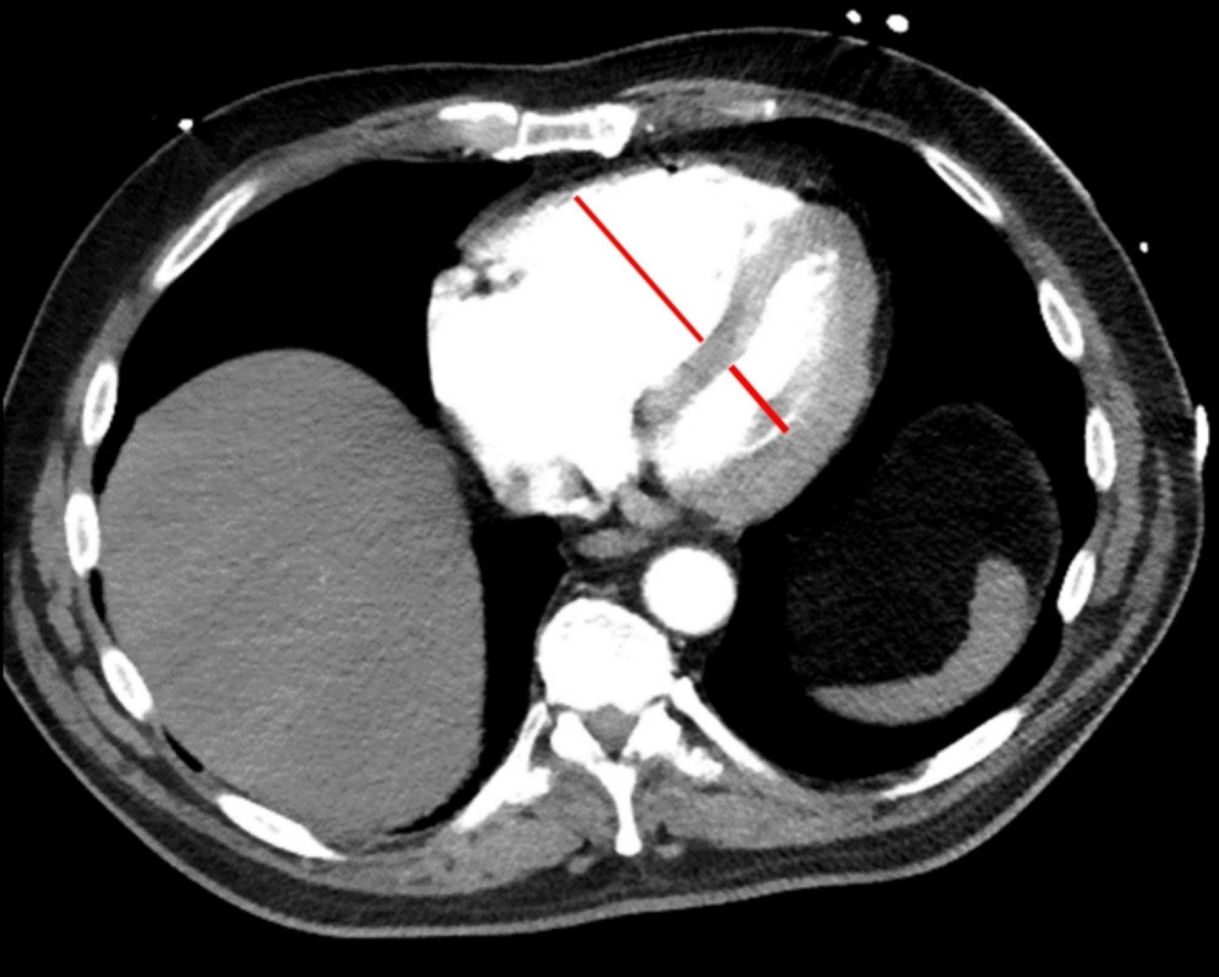
Measure of the RV/LV diameter ratio

**Fig. 2:**
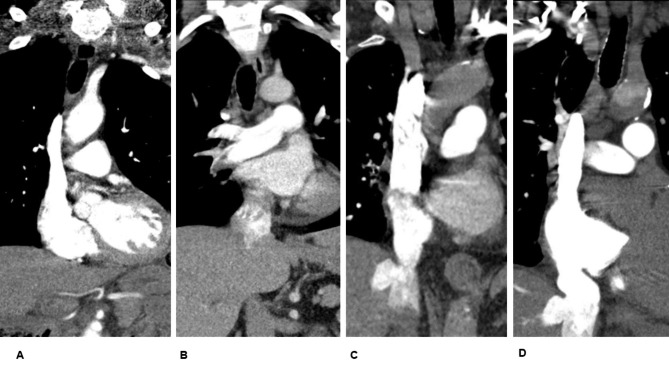
Estimation of the reflux into the inferior vena cava

For the purpose of the present study, the following radiological parameters were measured: the short-axis ratio of the right ventricle to the left ventricle (RV/LV), and the presence of contrast medium reflux into the inferior vena cava (IVC). The diameters of the right and left ventricles were estimated by identifying the largest points between the inner margins of the interventricular septum and the ventricle wall (Fig. 1). The reflux of contrast medium into the IVC was assessed on axial and coronal images, and it was quantified using a 4-point scale: no reflux (0 points), subcardial reflux into the IVC (1 point), intrahepatic reflux in the IVC (2 points), and subhepatic reflux in the IVC (3 points) (Fig. 2).

- No reflux into the inferior vena cava.
- Grade 1: Reflux into the suprahepatic inferior vena cava only.
- Grade 2: Reflux into the intrahepatic inferior vena cava as well and into the hepatic veins.
- Grade 3: Infrahepatic reflux.

All images were available in digital format and were analyzed on a PACS (picture archiving and communication system) dedicated workstation (Infinitt PACS, Version 3.0, Infinitt Healthcare, Korea). All images were analyzed in consensus by 2 radiologists with 3 and 20 years of radiological experience.

The following clinical-radiological scores were calculated (Table 2): BOVA score, pulmonary embolism mortality score (PEMS), European Society of Cardiology 2019 (ESC) score, Kumamaru score, and Calgary acute pulmonary embolism (CAPE) score [8–12]. Additionally, sPESI as a standard risk score was also calculated [13]. It is part of the risk stratification of the ESC guideline and PEMS.

**Table 2:** Analyzed combined clinical-radiological scores

Scores	Calculation
BOVA [8]	- Systolic Blood Pressure 90-100mmHg (2 points), - Cardiac troponin elevation (2 points), - RV dysfunction, echocardiogram or CT scan (2 points), - Heart rate ≥ 110 min^− 1^ (1 point).
PEMS [9]	- sPESI > 2 points (1 point), - pH < 7.35 (1 point), - minimal diastolic blood pressure < 45mmHg (1 point), - IVC reflux grade 3 (1 point), - need for vasopressors (2 points).
ESC 2019 [10]	- need for vasopressors, - sPESI ≥ 1, - RV dysfunction, echocardiogram or CT scan, - Cardiac troponin elevation.
Kumamaru [11]	- Age (years points), - Pleural effusion (20 points), - Pericardial effusion (20 points), - Lung nodule or liver mass being not apparently benign or multiple or destructive bone lesion (60 points), - History of chronic interstitial lung disease (20 points), - Enlarged lymph node of > 1 cm in diameter in the thorax (20 points), - Ascites (40 points).
CAPE [12]	- Heart rate ≥ 100 min^− 1^ (1 point), - Systolic Blood Pressure 90-100mmHg (1 point), - RV/LV ratio ≥ 1,5, CT scan (3 points), - Central PA clot (1 point)
sPESI [13]	- Age > 80 years old (1 point), - History of cancer (1 point), - History of chronic cardiopulmonary disease (1 point), - Heart rate ≥ 110 min^− 1^ (1 point), - Systolic Blood Pressure < 100mmHg (1 point), - Oxygen saturation < 90% (1 point).

### Statistical analysis

Statistical analysis was performed using SPSS (version 28, IBM SPSS Statistics for Windows, Armonk, NY, USA: IBM corporation). The collected data were evaluated by means of descriptive statistics (absolute and relative frequencies for categorical variables, means and standard deviations for continuous variables).

For every calculated score, sensitivity, specificity, negative and positive predictive values, Youden index, and area under the curve values were calculated for prognosis of 7- and 30-day mortality. For pairwise comparisons between the scores, kappa coefficients and McNemar test were reported. The in the literature suggested reported cut offs were used for the calculation.

## Results

Overall, 64 patients (12.0%) died and 467 patients (88.0%) survived within the 30-day observation time. 31 patients (5.8%) died within 7 days. The results of the analysed scores for survivors and non-survivors are shown in Tables 3 and 4. Furthermore, prognostic values of the scores to predict mortality were evaluated by using the reported cut-offs for every score. As shown, PEMS and CAPE had the highest specificity for 7-day mortality (93.4% and 85.0%, respectively). ESC 2019 showed the highest sensitivity (96.8%), followed by sPESI (90.3%). For 30-day mortality, PEMS and BOVA showed the highest specificity of 94.2% and 90.4%, respectively. The highest sensitivity was observed for ESC 2019 (95.3%), followed by sPESI (85.9%). All scores showed high negative predictive values and low positive predictive values for 7- and 30-day mortality.

The pairwise comparisons between the (dichotomized) scores show only moderate agreements (Table 5). Solely sPESI and ESC 2019 are an exception with a large kappa value of 0.834 (p < 0.001).

**Fig. 3:**
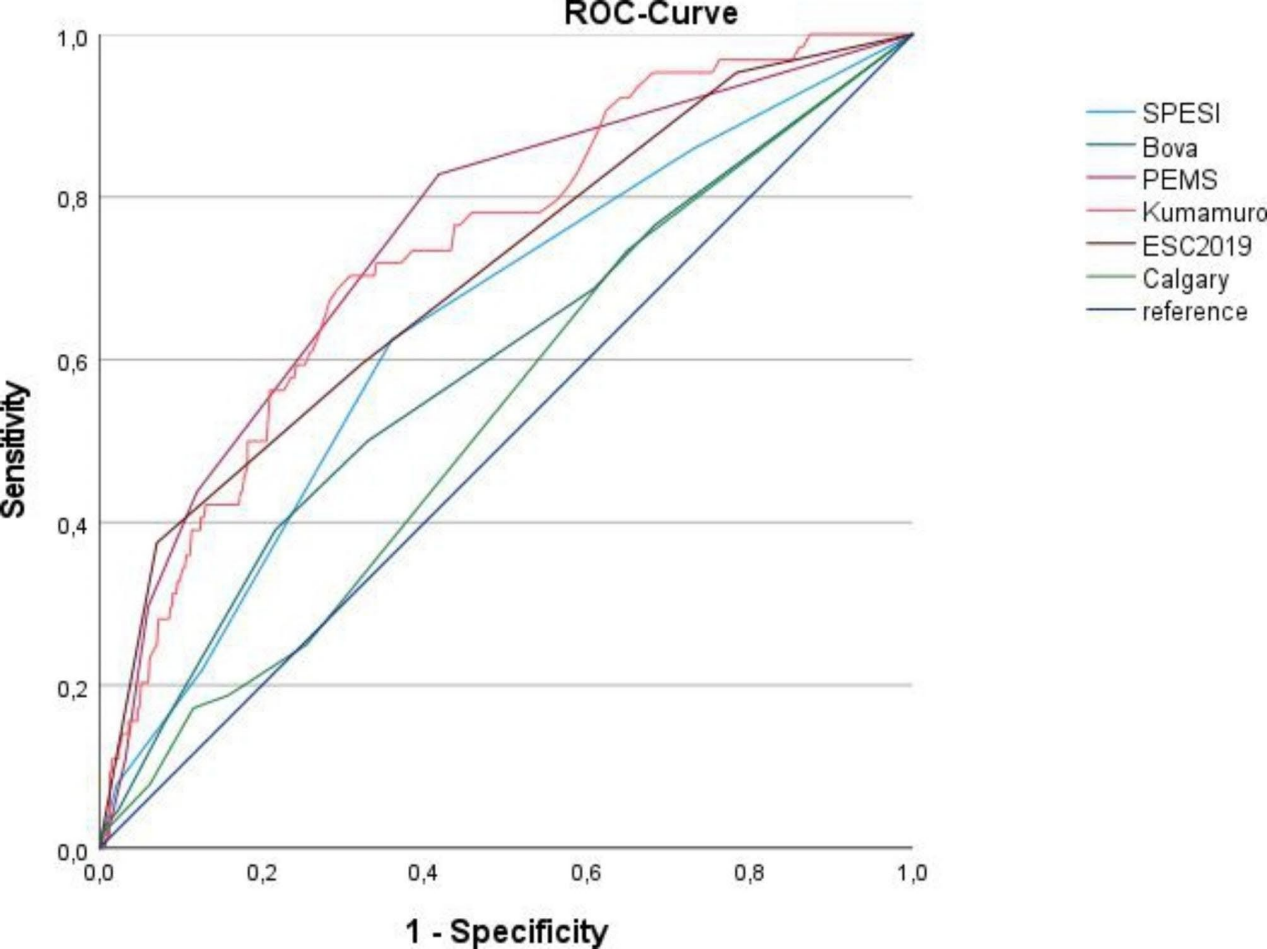
Receiver operating characteristic curves for the analyzed scores (30 day mortality)

Receiver operating characteristic curves for the analyzed scores are shown in Fig. 3 and Table 6.

**Table 3:** Predictive values for 7-day mortality in acute pulmonary embolism by using of different scores

Scores	Cutoff values	Sensitivity	Specificity	PPV	NPV	Youden index
sPESI	0 vs. >= 1	90.3%	26.6%	7.1%	97.8%	0.169
BOVA	<= 4 vs. > 4	54.8%	78.4%	13.6%	96.6%	0.332
PEMS	< 3 vs. >= 3	41.9%	93.4%	28.3%	96.3%	0.353
Kumamaru score	<=105 vs. > 105	74.2%	64.0%	11.3%	97.6%	0.382
ESC 2019 score	1 vs. >= 2	96.8%	20.8%	7.0%	99.0%	0.176
CAPE	< 3 vs. >= 3	35.5%	85.0%	12.8%	95.5%	0.205

**Table 4:** Predictive values for 30-day mortality in acute pulmonary embolism by using of different scores

Scores	Cutoff values	Sensitivity	Specificity	PPV	NPV	Youden index
sPESI	0 vs. >= 1	85.9%	27.2%	13.9%	93.4%	0.131
BOVA	<= 4 vs. > 4	39.1%	90.4%	20.0%	90.4%	0.295
PEMS	< 3 vs. >= 3	29.7%	94.2%	41.3%	90.7%	0.339
Kumamaru score	<=105 vs. > 105	70.3%	66.2%	22.2%	94.2%	0.365
ESC 2019 score	1 vs. >= 2	95.3%	21.8%	14.3%	97.1%	0.171
CAPE score	< 3 vs. >= 3	23.4%	84.8%	17.4%	89.0%	0.082

Abbreviations: sPESI: simplified pulmonary embolism severity index; PEMS: pulmonary embolism mortality score; ESC: European Society of Cardiology; CAPE: Calgary acute pulmonary embolism

**Table 5:** Pairwise comparison of the scores by kappa coefficient and McNemar test

	BOVA	PEMS	Kumamaru	ESC 2019	Calgary score
sPESI	0.102 (p_1_ < 0.001) (p_2_ < 0.001)	0.058 (p_1_ < 0.001) (p_2_ < 0.001)	0.284 (p_1_ < 0.001) (p_2_ < 0.001)	0.834 (p_1_ < 0.001) (p_2_ < 0.001)	0.068 (p_1_ = 0.007) (p_2_ < 0.001)
BOVA		0.217 (p_1_ < 0.001) (p_2_ < 0.001)	0.088 (p_1_ = 0.032) (p_2_ < 0.001)	0.141 (p_1_ < 0.001) (p_2_ < 0.001)	0.361 (p_1_ < 0.001) (p_2_ < 0.001)
PEMS			0.032 (p_1_ = 0.278) (p_2_ < 0.001)	0.046 p_1_ < 0.001) (p_2_ < 0.001)	0.095 (p_1_ = 0.020) (p_2_ < 0.001)
Kumamaru score				0.232 (p_1_ < 0.001) (p_2_ < 0.001)	0.028 (p_1_ = 0.449) (p_2_ < 0.001)
ESC 2019 score					0.059 (p_1_ = 0.001) (p_2_ < 0.001)

p_1_ = p-value of the kappa coefficient

p_2_ = p-value of the McNemar test

**Table 6:** Receiver operating characteristic curves for the analyzed scores (30-day mortality)

	AUC	Standard error	p-value	95% confidence interval	
				Lower bound	Upper bound
SPESI	0.641	0.037	< 0.001	0.569	0.713
Bova	0.593	0.040	0.019	0.515	0.672
PEMS	0.750	0.033	0.000	0.685	0.814
Kumamuro	0.735	0.032	0.000	0.672	0.798
ESC	0.704	0.036	0.000	0.633	0.774
Calgary	0.537	0.037	0.327	0.463	0.610

## Discussion

Risk estimation in acute pulmonary embolism (APE) is crucial due to its high mortality rate. Several scores are used to stratify APE, with the simplified pulmonary embolism index (sPESI) being commonly employed in clinical practice.

sPESI is a clinical score that consists of six equally weighted variables, each assigned a point: age > 80 years, presence of cancer, chronic heart failure or chronic pulmonary disease, systolic blood pressure < 100mmHg, and arterial oxyhemoglobin saturation < 90% [13]. This score effectively distinguishes patients with a low risk of mortality (0 points) [13]. However, the literature suggests that sPESI fails to identify patients with intermediate risk, such as those with right ventricular dysfunction (RVD) and/or elevated cardiac biomarkers [10]. This suggests that the precision of sPESI can be enhanced by incorporating signs of right heart injury. In the ESC 2019 guidelines, in addition to sPESI (≥ 1 point), the need for vasopressors, signs of RVD on echocardiography or computed tomography, and cardiac troponin elevation are included [10]. ESC has been validated in numerous studies and exhibits high accuracy. For instance, it can predict adverse events in APE with a sensitivity of 96% (95% CI 79–99) and a negative predictive value of 99% (95% CI 95–99), while its specificity and positive predictive value are 57% (95% CI 53–62) and 11% (95% CI 8–17), respectively [14]. This is mirrored in the present analysis analysis focusing on the prediction of 7-day and 30-day mortality. ESC shows a high sensitivity (96.8% and 95.3%, respectively) and negative predictive value (99.0% and 97.1%, respectively), but low specificity and negative predictive value.

PEMS is another combined score that incorporates sPESI, clinical parameters, and radiological signs of RVD. This score includes sPESI (< 2 points), pH values, clinical indicators (minimal diastolic blood pressure, need for vasopressors), and radiological signs of RVD (IVC reflux grade 3). In the original study, PEMS demonstrated the following diagnostic values: sensitivity 84.9%, specificity 83.0%, positive predictive value 51.8%, and negative predictive value 96.2% [8]. The present study is the first validation of PEMS, showing that it exhibits the highest specificity (93.4% and 94.2% for 7- and 30-day mortality, respectively) in the cohort, along with a high negative predictive value (96.3% and 90.7%, respectively).

The BOVA score is a model that solely incorporates clinical and radiological signs of RVD [9]. It demonstrates a high area under the curve value for predicting in-hospital death (0.908 [15]. The BOVA score has been validated in numerous studies. According to a meta-analysis, the pooled sensitivity, specificity, positive likelihood ratio, and negative likelihood ratio of the BOVA score for predicting short-term composite adverse outcomes are 0.25 (95% CI, 0.22–0.29), 0.93 (95% CI, 0.92–0.93), 4.05 (95% CI, 2.90–5.67), and 0.81 (95% CI, 0.74–0.88), respectively [16]. Our results align with the reported data, indicating a moderate specificity for 7-day mortality (78.4%), but a good for 30-day mortality (90.4%) and a high negative predictive value (96.6% and 90.4%, respectively) for the BOVA score. Its observed sensitivity and positive predictive value are low.

The Kumamaru score primarily relies on radiological findings and incorporates multiple features, such as pleural effusion, pericardial effusion, and enlarged lymph nodes in the thorax [11]. According to Kumamaru et al., it exhibits a high area under the curve value for predicting 30-day mortality (0.89) [11]. However, there have been no investigations validating this score to date. In our cohort, the Kumamaru score demonstrates low sensitivity, specificity, and positive predictive value for both 7- and 30-day mortality.

The CAPE score is a recently published model that incorporates clot burden, as well as radiological and clinical signs of RVD [12]. Unfortunately, the prognostic values (sensitivity, specificity) of this score regarding mortality are not yet known. Our study represents the first validation of the CAPE score, indicating low sensitivity (35.5% and 23.4%, respectively) and moderate specificity (85.0% and 84.8%, respectively) in our cohort.

To the best of our knowledge, this is the first study to compare the diagnostic performance of different clinical-radiological scores reported for predicting 7- and 30-day mortality in APE. Overall, our findings demonstrate that some of the proposed scores exhibit low sensitivity and/or specificity, while all scores display low positive predictive values. However, all scores demonstrate high negative predictive values ranging from 95.5 to 99.0% for 7-day and 89.0–97.1% for 30-day mortality.

In the absence of a standardized definition for PE-related death, we chose all-cause mortality as a reasonable endpoint to express consequences of PE in patients. We chose all-cause 7- and 30-day mortality as the primary outcome. Jimenez et al. have shown in an analysis of the RIETE registry that 7-day all-cause mortality is close to 7-day PE-related mortality (1.8% and 1.1% for the time frame 2010–2013) [17]. The gap was wider for 30-day mortality, with 4.9% all-cause mortality and 1.8% PE-related mortality. PE-related mortality was defined as deaths confirmed by autopsy or those following severe PE. In the absence of an alternative diagnosis, death was judged as due to fatal PE. A similar gap for mortality was found in the EMPEROR study [18].

The present study has some limitations. It was a retrospective study in a monocenter setting. It is possible that patients in a critical clinical condition did not undergo CT-scans, leading to selection bias. The diagnosis of APE was based on CT-scans only. Patients with contraindications to radiation or contrast medium were excluded from our cohort. However, our results are based on a large cohort.

In conclusion, for prognosis 7- and 30-day mortality in APE, PEMS score has the highest specificity. ESC 2019 shows the highest sensitivity. All scores had low positive and high negative predictive values.

## Data Availability

Data can be assessed upon reasonable request to the corresponding author.
